# Characterization of Microbiome Diversity in the Digestive Tract of *Penaeus vannamei* Fed with Probiotics and Challenged with *Vibrio parahaemolyticus* Acute Hepatopancreatic Necrosis Disease

**DOI:** 10.3390/pathogens14040320

**Published:** 2025-03-27

**Authors:** Lucio Galaviz-Silva, Abraham O. Rodríguez de la Fuente, Ricardo Gomez-Flores, José C. Ibarra-Gámez, Itza Eloisa Luna-Cruz, Joel H. Elizondo-Luevano, Ricardo Sánchez-Díaz, Zinnia J. Molina Garza

**Affiliations:** 1 Universidad Autónoma de Nuevo León, Facultad de Ciencias Biológicas, Laboratorio de Patología Molecular y Experimental, San Nicolás de los Garza 66455, Nuevo León, Mexico; lucio.galavizsl@uanl.edu.mx; 2 Universidad Autónoma de Nuevo León, Facultad de Ciencias Biológicas, Departamento de Ciencias Exactas y Desarrollo Humano, San Nicolás de los Garza 66455, Nuevo León, Mexico; abraham.rodriguezd@uanl.edu.mx; 3 Universidad Autónoma de Nuevo León, Facultad de Ciencias Biológicas, Departamento de Microbiología e Inmunología, San Nicolás de los Garza 66455, Nuevo León, Mexico; rgomez60@hotmail.com (R.G.-F.); itza.lunacrz@uanl.edu.mx (I.E.L.-C.); 4 Instituto Tecnológico de Sonora, Departamento de Ciencias Agronómicas y Veterinarias, Ciudad Obregón 85000, Sonora, Mexico; ricardos_d@hotmail.com; 5 Facultad de Farmacia, Instituto de Investigación Biomédica de Salamanca, Campus Miguel de Unamuno s/n, Universidad de Salamanca, 37007 Salamanca, Spain; jelizondo@usal.es

**Keywords:** probiotics, digestive tract, shrimp, high throughput sequencing, *Bacillus pumilus*, *Vibrio alginolyticus*, *V. campbellii*

## Abstract

The microbiome of the shrimp’s digestive tract shows differences between healthy and acute hepatopancreatic necrosis disease (AHPND)-affected shrimp. The present study aimed to evaluate the impact of probiotic consumption on the microbial community in experimentally AHPND-infected shrimp. Effective probiotics (EPs) *Vibrio alginolyticus* (Va32A), *V. campbellii* (VcHA), and *Bacillus pumilus* (BPY100) and non-effective probiotics (NEPs) *B. pumilus* (Bp43, and BpY119), were employed in bioassays with *Penaeus vannamei* and challenged with AHPND-causing *V. parahaemolyticus* (Vp_AHPND_). Stomach (Sto), intestine (Int), and hepatopancreas (Hep) were analyzed by metabarcoding (16S rRNA gene) to characterize the microbiome and biomarkers. Hep-VcHA showed the highest alpha diversity (Shannon index = 5.88; 166 ASVs), whereas the lowest was for Hep-Bp43 (2.33; 7 ASVs). Proteobacteria, Actinobacteria, Bacteroidetes, and Saccharibacteria were the most abundant phyla. The relative abundance of *Vibrio* sp. was the highest in the Hep and Int of Bp43, BPY119 and the positive control, followed by *Rhodobacteraceae* in the EP group. Principle coordinate analysis (PCoA) showed a cluster grouped negative (Sto and Hep) control with almost all organs in the EP group causing 28.79% of the variation. The core microbiome of EP was mainly represented by *Rhodobacteraceae*, Caldilineaceae, *Celeribacter indicus*, *Illumatobacter*, *Microbacterium*, *Ruegeria atlantica, Saccharibacteria* sp., *Shimia biformata*, and *Thalassobius mediterraneus*, whose relative abundance was enriched by probiotics, which may explain their protective roles against Vp_AHPND_, whereas the low survival in the NEP group was associated with a higher diversity of *Vibrio* spp. Our results present an ecosystem-friendly alternative based on beneficial microorganisms to prevent and control AHPND and probably other bacterial diseases in shrimp farming.

## 1. Introduction

*Penaeus vannamei* is the most widely farmed shrimp in Asia and Latin America, with a yearly production of 5.6 million metric tons [1]. However, the shrimp industry is continuously exposed to infectious diseases, which emerge and spread worldwide, resulting in significant economic damage [2]. Acute hepatopancreatic necrosis disease (AHPND), also named Early Mortality Syndrome (EMS), is a recent affliction originally caused by virulent *Vibrio parahaemolyticus* (Vp_AHPND_) harboring the pVA1 plasmid (63 kb to 70 kb), which encodes the binary PirA^VP^/PirB^VP^ toxins [2]. This pathogen rapidly emerged in Mexico at the beginning of 2013 [3] and outbreaks have amounted to a global loss of over USD 43 billion to the shrimp-producing countries [4]. Bacterial diseases, including AHPND, are commonly treated with antibiotics. However, their extensive misuse leads to the development of resistant or multi-drug-resistant strains. In Mexico, Vp_AHPND_ strains resistant to oxytetracycline and sulfachloropyridazine–trimethoprim have been found [5]. Moreover, antibiotics remove beneficial bacteria thriving in the digestive tract. This microbiota may function as probiotics due to its beneficial functions for healthy development, providing considerable enzymatic potential that supports nutrient digestion, synthesis of vitamins, growth regulation, and immune defense against pathogenic bacteria [6,7]. Previous studies have evaluated the microbiota in the digestive tract of healthy *P. vannamei* [8,9] or its response to Vp_AHPND_ in several aquatic environments [10,11,12,13].

Vp_AHPND_ colonizes in the stomach as an initial stage of infection and the cellular damage caused by its toxin is followed by hepatopancreas colonization [10,14]. The stomach microbial diversity has a crucial function in protecting against bacterial infections. Its diversity is broader in the stomach, where the phyla Proteobacteria, Firmicutes, and Bacteroidetes predominate [15]. Among them, *Rhodobacteraceae* (Alphaproteobacteria) plays an important role as a first defense barrier against pathogenic bacteria [16]. Regarding the gut microbial community, the phyla Proteobacteria, Bacteroidetes, and Actinobacteria form the intestinal core microbiome and are present through all growth stages in healthy *P. vannamei* [17,18]. However, the digestive tract microbiome has differences between healthy and AHPND/EMS-diseased shrimp, in which a progressive shift in the gut microbiota diversity enriched with *Photobacterium* and *Vibrio* occurs, changing or reducing the bacterial diversity, leading to dysbiosis [11,19].

Scarce information is available on the effects of probiotics on the shrimp digestive tract microbiota during Vp_AHPND_ challenges; probiotic treatments with *Lactobacillus casei* (P1), *Rhodopseudomonas palustris* (P2), a combination of two microorganisms (P3), or a commercial probiotic (EM) resulted in shrimp survival rates of 11.7%, 26.7%, 36.7% and 73.3% (P1, P2, P3, and EM, respectively); those with lower survival (P1–P3) showed greater relative abundance of *Vibrionaceae* (60%) in the gut and the bacterial diversity (Shannon indices) values decreased significantly in P1–P2 and P3 (i.e., 4.12 ± 0.058 to 0.17; *p* < 0.05), but in contrast, it increased in shrimp treated with EM and the negative control [20]. In addition, an ILI probiotic (*Vibrio diabolicus*) was shown to control infection in shrimp after being challenged with Vp_AHPND_. Shannon’s index demonstrated a high diversity in shrimp treated with the probiotic. Moreover, analysis of the microbial community structure demonstrated that Proteobacteria, Firmicutes, and Tenericutes were the most abundant phyla, but a reduction in the number of phyla occurred in the hepatopancreas and stomach of Vp_AHPND_-infected shrimp [21]. *Bacillus subtilis* is known to harbor at least two AMP-related genes that inhibit Vp_AHPND_ [22].

The present study is closely related to our previous report showing the antagonistic activity of probiotic bacteria 32A, Y100, HA, Y119, and 43 against Vp_AHPND_ in bioassay challenges with *P. vannamei*. The 32A, Y100, HA bacterial strains significantly reduced the cumulative mortality as effective probiotics (EP, 4.76%, 12.54%, and 16%, respectively) and all challenges were Vp_AHPND_-qPCR negative, showing their microbicidal effect, whereas the non-effective (NEP) Y119 and 43 did not show a protective role [23]. Because of the limited information regarding the impact of probiotic treatments on the bacterial composition of the shrimp digestive tract challenged with VP_AHPND_, this work aimed to evaluate the profile of microbiome dynamics promoted by the effects of feeds supplemented with EPs in comparison to those supplemented with NEP strains.

### Ethics Statement

All experimental procedures were approved by the Animal Ethics Committee for Laboratory Animals from Universidad Autónoma de Nuevo León, México (Registration number CEIBA-2023-014), and did not involve endangered or protected species.

## 2. Materials and Methods

### 2.1. Experimental Design

This metagenomic study included seven bioassays with three replicate determinations, five with probiotics that had been recognized as antagonists for the pathogenic VP_AHPND_. The strains are labeled here as Va32A, BpY100, VcHA, BpY119, and Bp43, and the positive and negative controls as Pos and Neg, respectively. Isolates Va32A and VcHA (collected from *Rhizophora mangle* and mangle mud) corresponded to *Vibrio alginolyticus* and *V. campbellii*, respectively, and Bp43, BpY100, and BpY119 matched with *Bacillus pumilus* strains, isolated from Chinese clam, saline sediment, and seawater, respectively. A detailed description of the bioassay using probiotic bacteria with their identity and GenBank accession number was previously reported [23]. In brief, the experiment was conducted in a climate-controlled room using an indoor recirculating clear water system. Shrimp (kindly donated by Megalarva de Sinaloa, S. de R.L.) were acclimatized in a fiberglass tank with optimal conditions. Water parameters were monitored, including salinity (35 g/L), dissolved oxygen (~5–6 ppm), temperature (28–30 °C), and pH (7.6–8.2). Treatments were applied to 20 shrimp (2 g average weight) per group in disinfected aquaria of 30 L. Probiotic diets were prepared with fresh bacterial cultures (BpY100, BpY119, Bp43, Va32A, and VcHA) in trypticase soy broth + 2% NaCl (TSB-N; Difco, Becton Dickinson, Franklin Lakes, NJ, USA), incubated at 30 °C for 24 h. Next, the bacterial pellet was precipitated by centrifugation and resuspended in phosphate-buffered saline solution (0.5 to 0.6 McFarland standard; 1.5 × 10^8^ CFU/mL) and 300 mL of this suspension was added per kilogram of commercial shrimp feed (Rangen, Inc., Buhl, ID, USA) by spraying and orally administered twice daily at 3% body weight with their respective diet for two weeks before the experimental challenge. Experimental infection was then performed by immersing the shrimp in a suspension of the pathogen VP_AHPND_ in TSB-N (8 × 10^5^ CFU/mL) for 15 min, with aeration supplemented by an air pump, returned to the aquaria and fed daily with their respective probiotic dosage. The positive control (Pos) was infected using the same method, but without the probiotic. The negative control (Neg) was immersed in sterile TSB-N [23].

### 2.2. Sampling of Stomach (Sto), Intestine (Int), and Hepatopancreas (Hep) for Metagenomic Analysis

Five lethargic (positive control, which showed the clinical signs of AHPND, including a pale hepatopancreas and empty intestine) or healthy shrimp from each aquarium were randomly collected (15 from each treatment), washed with 75% ethanol, and rinsed with sterile saline solution in a laminar-flow cabinet under aseptic conditions. Sto, Int, and Hep samples were aseptically collected in DNA/RNA Shield reagent (ZymoBIOMICS; Zymo Research, Irvine, CA, USA). Each type of organ was separately pooled and used for microbiome analyses, giving a total of 21 samples. Labels for each region of the digestive tract and probiotics were prepared with the name of the organ first, followed by the probiotic strain (e.g., Sto-BpY100).

### 2.3. DNA Extraction, Library Preparation, Sequencing, and Bioinformatics Analyses

Production of the 16S rDNA library, sequencing, raw read curation, and microbial community analysis were processed by the ZymoBIOMICS DNA extraction method. Total DNA was extracted from the organs with a DNA Miniprep Kit and quantified with a Qubit fluorometer (Life Technologies, Carlsbad, CA, USA). Bacterial 16S ribosomal RNA gene-targeted sequencing was performed using the Quick-16S NGS Library Prep Kit V3-V4, following the supplier’s instructions (Zymo Research). In brief, amplicons were prepared using 100 ng of the total DNA from the samples. Targeted library preparation was performed with the master mix reactions for the 1-Step PCR for the bacterial 16S ribosomal RNA gene-targeted sequences. The Quick-16S™ Primer Set V3-V4 341f (CCTACGGGDGGCWGCAG, CCTAYGGGGYGCWGCAG) and 806r (GACTACNVGGGTMTCTAATCC) (Zymo Research) amplifies the V3–V4 region of the 16S rRNA gene. PCR reactions were adjusted to a final volume of 30 μL and performed in a real-time PCR (qPCR) thermocycler to control the cycles. The final qPCR amplicon with index sequences of 606 bp was confirmed and quantified with the qPCR fluorescence-based method (Qubit dsDNA HS Assay kit; Thermo Fisher Scientific, Inc., Waltham, MA, USA), and read and pooled based on equal molarity. The final pooled library was cleaned and quantified [TapeStation (Agilent Technologies, Santa Clara, CA, USA) and Qubit (Thermo Fisher Scientific)]. The final libraries were subjected to 2 × 150 bp sequencing on the Illumina MiSeq platform with the Miseq v3 reagent kit (600 cycles). The sequencing was performed with 10% PhiX spike-in, following the manufacturer’s instructions (Illumina, Inc., San Diego, CA, USA).

### 2.4. Bioinformatic Analysis

Unique amplicon sequence variants (ASVs) were inferred from raw reads, and the sequences of primers, barcodes, chimeric sequences, and adaptors were trimmed, denoised, and filtered with the DADA2 pipeline [24]. Sequence data were analyzed using Uclust from Quantitative Insights into Microbial Ecology (Qiime v.1.9.1), after which taxonomy assignment to ASVs was performed using Uclust and a 16S database from Zymo Research, internally designed and curated, as a reference. Composition visualization, bar plots of relative abundance, alpha diversity (species richness, species diversity, Chao1, and Shannon index, respectively), and beta diversity analyses were performed with Qiime v.1.9.1 [25]. Principal coordinate analyses (PCoA) plots were performed with internal scripts, based on Bray–Curtis distances and visualized using three-dimensional plots in EMPeror (a part of QIIME). The ASV abundance heatmap was directly designed from the abundance of unique amplicon sequences inferred from the raw sequencing data and showed the microbial composition of the samples with the top fifty most abundant species identified. Hierarchical clustering was performed on samples based on the Bray–Curtis dissimilarity Dada2 pipeline [24].

We analyzed significant differences in the microbiota from digestive tract fractions, using the non-parametric Kruskal–Wallis test with Bonferroni correction for multiple comparisons, considering *p* < 0.05 as significant. LEfSe analysis was performed to identify biomarkers (taxa with significantly different distributions among treatment groups; *p* < 0.05) with an effect size (LDA score) greater than two [26].

### 2.5. Quality Controls

The Microbial Community DNA Standard (Zymo Research) was used as a positive control for DNA extraction and each targeted library preparation. For negative controls (blank extraction control and blank library preparation), we did not add DNA templates to the reactions.

## 3. Results

### 3.1. Read Processing and Alpha Diversity

We generated 1,135,096 raw read sequences (R1 + R2). After trimming and size filtration of 1,133,166 sequences, we obtained 1930 final unique ASVs identified as the observed species. Bacterial richness among the different organs in general ranged from 7 (Hep-Bp43) to 166 (Hep-VcHA) ASVs. Regarding the total final unique sequences per probiotic trial, the highest ASV number was shown for VcHA (449), followed by the Neg (293), Pos (281), Va32A (275), BpY100 (223), BpY119 (213), and Bp43 (196) with the lowest richness (Table 1).

**Figure 1:**
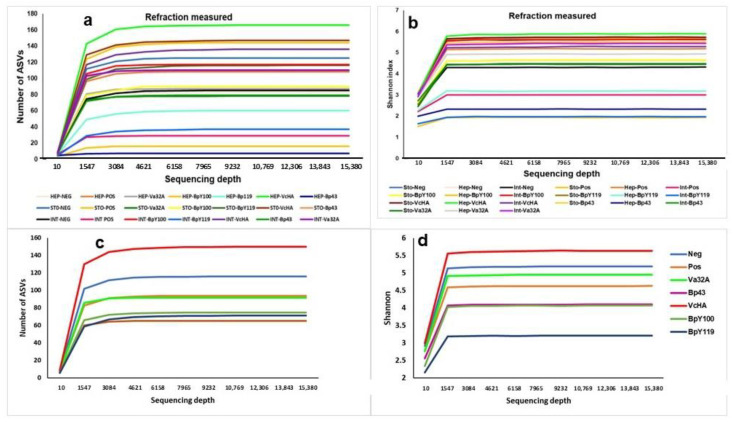
Refraction plots of bacterial richness (**a**) and Shannon index (**b**) among Int, Hep, and Sto, and the different challenges (**c**,**d**). Int showed significant (*p* < 0.0001) differences between the means in the different challenges in the analysis of Kruskal–Wallis with post hoc Bonferroni correction, highlighting differences (*p* < 0.0001) among Pos with Va32A, BpY100, and VcHA. Abbreviations: Int = intestine, Hep = hepatopancreas, Sto = stomach.

The alpha diversity rarefaction plots (Figure 1a–d) at a 15,380-sequencing depth show that the number of observed species reaches a *plateau* at around 3084–4621 and the Shannon index at 1547 of the sequencing depth. Significant differences (*p* < 0.0001) were found in the Shannon index among the organs (Figure 1a,b) and different probiotic trials (Figure 1c,d). The results showed sufficient sequencing depth by a *plateau* for each sample, demonstrating that sequencing captured most of the abundant bacterial species and further sequencing would not lead to the detection of new bacterial species. Concerning the comparative alpha diversity by organs, the highest corresponded to Hep-VcHA (Shannon index = 5.88), followed by Sto-VcHA (5.731), Sto-Pos (5.69), and Sto-Neg (5.62), and the lowest was for Hep-BpY100 (1.94), Int-BpY119 (1.974), and Hep-Bp43 (2.335) (Figure 1b). Alpha diversity for the different trials showed the highest number of observed species and Shannon index for VcHA, followed by Va32A, whereas the lowest metric was for the NEP, Bp43, and BpY119 (Figure 1c,d). Rarefaction curves were obtained using only those sequences assigned to a species as an estimate of diversity at that taxonomic level.

**Figure 2:**
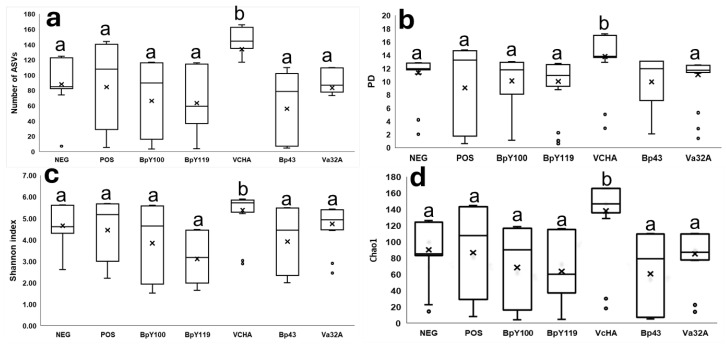
Alpha diversity of the microbiota of *Penaeus vannamei* fed with five different probiotics and challenged with VpAHPND. Individual values are shown, and the box delimits the 25th and 75th percentiles. The line in each box indicates the median and the whiskers indicate the lowest and highest values. Columns not connected by the same letter indicate that the means were significantly different according to Tukey’s HSD test (*p* < 0.05). Comparative statistical analysis with the Kruskal–Wallis test with Bonferroni correction among the trials, showed the number of ASVs (**a**), phylogenetic diversity (PD; (**b**)), Shannon index (**c**), and Chao1 (**d**).

We observed significant (*p* < 0.01) differences in diversity and richness among the Pos with probiotics Va32A, BpY100, and VcHA by the Kruskal–Wallis test followed by Bonferroni post hoc analysis (Figure 1a,b) and confirmed that VcHA is highlighted as the probiotic with the highest richness and diversity (Figure 2a–d). In addition, the Spearman correlation test confirmed that the high alpha diversity of these three strains (Shannon index > 4.6) was associated (rho = −0.70) with a low mortality rate (<1.6%), whereas the NEP, with a high mortality rate (>70%), had a low alpha diversity (Shannon index < 4).

Int-BpY119 showed statistical significance against Va32A (*p* < 0.05), BpY100 (*p* < 0.002), VcHA (*p* < 0.005), and Bp43 vs VcHA (*p* < 0.005). Sto also showed significant differences (*p* < 0.001) between Va32A with Pos, Neg, and VcHA, as well as between BpY100 with Pos and VcHA, and Bp43 with VcHA. Furthermore, the Hep richness presented statistical differences for Bp43 with Neg, Pos, and Va32A; BpY100 with Pos; and BpY119 against VcHA (*p* < 0.0001) (Figure 1a–d).

The microbial community structure among the five bioassays and controls indicated that Proteobacteria, Actinobacteria, Bacteroidetes, and Saccharibacteria were the most abundant phyla among Hep, Int, and Sto. The accumulated abundance reached up to 99.6% for Proteobacteria, followed by Bacteroidetes (33.2%), Actinobacteria (31.8%), and Saccharibacteria (5.5%) (Table 2).

According to the ASVs, sequence analysis at the phylum level revealed that the most abundant phyla in the challenges and controls were Proteobacteria in the 21 trials, from 48.7% (Hep-VcHa) to 99.6% (Int-Pos). Actinobacteria and Bacteroidetes were distributed in 19 trials (0% to 31.8% and 0% to 20.6%, respectively), and Chloroflexi in 17 (0% to 7.3%). The phyla with the lowest abundance were Fusobacteria (Pos-Hep and Sto), Gemmatimonadetes (Pos-Sto and VcHA-Hep), and Spirochaetae (Pos-Hep and Sto), ranking from 0% to 0.2%, whereas Lentisphaerae (VcHA-Hep, 0.1%), Chlamydiae (VcHA-Sto, Hep, and Int, 0.1%), and Cyanobacteria Chloroplast (BpY100-Sto, 0.1%) occurred in only one probiotic trial. Sequences that were not taxonomically classified were labeled as “None: others”, and they showed the highest frequency in Neg (Hep and Int) and Bp43 (Int) (Supplementary Tables S1 and S2). Details of the relative frequency of the 15 phyla and 28 classes are available in Supplementary Tables S1 and S2.

According to the analyses at the class level, Gammaproteobacteria exhibited a wide distribution in all trials (844.3% of the cumulative frequency), from 0.7% (Hep-Neg) to 99.2% in Int-Pos, showing the highest abundance. However, it is important to underline that the other classes were reduced, since only Actinobacteria, Clostridia (Firmicutes), and Alphaproteobacteria were present with a low relative frequency (0.3%, 0.1%, and 0.3%, respectively). Hep-Bp43 also showed a low diversity in their bacterial community, since only two phyla were present, Proteobacteria (82%), represented by the classes Alphaproteobacteria (0.1%), Deltaproteobacteria (22.5%), and Gamaproteobacteria (59.5%); and the phylum Saccharibacteria at 18% (Supplementary Table S2). This was followed by Alphaproteobacteria (0.1% to 59.6%) (Supplementary Table S2).

Gamaproteobacteria also reached the highest frequency in Hep-BpY100 with 97.7%, whereas Hep-Neg (0.7%) and Sto-BpY100 (1.5%) showed the lowest abundance. Gamaproteobacteria was followed by Alphaproteobacteria (cumulative frequency of 540.9%), which reached 59.6% in Hep-Neg and 56.9 in Sto-BpY100, but only 0.1% in Hep-Bp43 (Supplementary Table S2).

**Figure 3:**
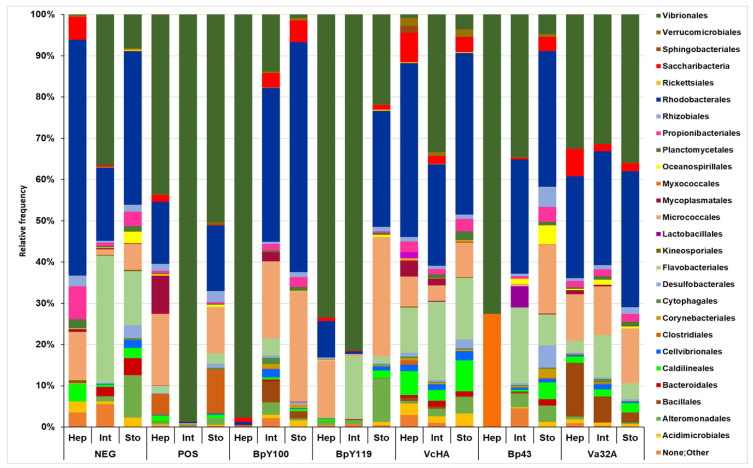
Relative abundance at the order level of the microbiome composition in the five probiotic trials in shrimp experimentally infected with AHPND-causing *Vibrio parahaemolyticus*. For abbreviations see Figure 1.

Regarding the sequence analysis at the order level, Vibrionales (Gamaproteobacteria), Rhodobacterales (Alfaproteobacteria), and Micrococcales (Actinomycetes) were the most prominent in almost all trials. Vibrionales reached the highest abundance in Int-Pos, (98.7%), Hep-BpY100 (97.7%), and Int and Hep-BpY119 (80.9% and 73.5%, respectively). Regarding Neg, the abundance in these organs was significantly lower at 0.2% to 36.3%. In almost all trials of probiotics, Hep or Int shared the greatest abundance of Vibrionales. In contrast, Rhodobacterales were more frequent in Hep, Int, and Sto of Neg (56.8%, 17.7%, and 35%). It was absent only in Hep of Bp43. Abundance in the microbiome was followed by Micrococcales (Actinomycetes) and Flavobacteriales (Bacteroidia), with a presence in 19 of 21 trials, the former one in Sto and Int-BpY100 (26.8% and 18.4%), and BpY119 (Sto, 28.6%, and Hep, 13.8%). Flavobacteria reached their highest abundance in Int-Neg (30.8%) and Sto-Neg (12.3%) in comparison with Pos, which ranged between 0% to 2.2%; the VcHA trial also increased its abundance from 10.4% to 18.7% (Figure 3).

The relative frequency of the main genera and species of Sto, Int, and Hep microbiome were significantly (*p* ≤ 0.0001) different in all shrimp trials. In addition, the relative abundance of *Vibrio* sp. indicated that it was the most frequent bacteria in all organs (cumulative abundance of 331.4%) among the seven bioassays (0.1% to 81.1%), reaching the maximum frequency in the Hp-Y100 trial (81.1%), followed by that of Hep-Bp43 (59.5%) and Hep-BpY119 (54.3%). In controls of Int-Pos, the abundance was 25.6%, and 12.2% in the Neg-Int, whereas the lowest abundances (0.1%, 0.2%, and 0.4%) corresponded to Hep-Neg, Int-VcHA, and Sto-BpY100. The abundance of *Vibrio* sp. was followed by that of *Rhodobacteraceae* (cumulative abundance of 174.1%), with the highest frequencies in Hep (31.3%), Int (4.5%), and Sto (18.5%) of Neg, followed by Pos (0% to 4.3%). *Vibrio shilonii* was mainly abundant in Int-BpY119 (79%), Int-VcHA (25.4%), Int-Neg (19.1), and Bp43 (16.7%), and *Spongiimonas flava* (Bacteroidetes) and *V. shiloni* were the most abundant representatives in Hep and Int-Neg, along with *Shimia biformata* (Alphaproteobacteria, Rhodobacterales) in Hep-Neg. *Saccharibacteria* was also present in almost all probiotic trials, with a minimum frequency of 0.10% in the Int-BpY119 and 18% in the Hep-Bp43 probiotic trials. This phylum was absent only in Int-Pos. *Vibrio parahaemolyticus* (non-AHPND) was identified in the positive control (0.2%), Sto of Y119 (0.4%), Bp43 (0.1%), and in the Sto and Int of Va32A (Supplementary Table S3).

Regarding the representative distribution of the microbiome among the controls and probiotic challenges in Pos, the most predominant bacteria in Int, Hep, and Sto were *Vibrio alginolyticus* (57.9%, 9.3%, and 10.2%, respectively), *Vibrio* sp. (25.6%, 18.7%, and 13.6%, respectively), and *Photobacterium damselae* (7.9%, 8.8%, and 13%, respectively). *Brachybacterium paraconglomeratum* was also present in Hep, Sto, and Int (12.6%, 8%, and 0.3%, respectively), which highlights the difference from Neg (0.4%, 0%, and 0.2%, respectively), where Int showed the absence of this species (Supplementary Table S3).

Probiotic BpY100 increased the abundance of *Celeribacter indicus* up to 11.4% in Int and 19% in Sto, along with *B. paraconglomeratum* (9.7% and 14%) and *Rhodobacteraceae* (8.1% and 13.3%) in Hep of BpY100 *V. harveyi* (16.6%), followed by *Vibrio* sp. Probiotic Bp43 in Hep increased the abundance of *Sandaracinaceae* (22.5%) and *Saccharibacteria* sp. (18%), and in Int *V. shilonii*, it increased the abundance of *Shimia* (17.5%) and *Spongiimonas flava* (14.7%). The most frequent bacteria in Hep of Bp119 were *Vibrio* sp., *V. alginolyticus*, and *B. paraconglomeratum* (54.3%, 19.1%, and 13.2%, respectively), and in Int they were *V. shilonii* (79%) and *Spongiimonas flava* (15%). Va32A was a common member in Sto, Int, and Hep for *Vibrio mytili* (20% to 34.1%), Rhodobacteraceae (9.3% to 13%), and *B. paraconglomeratum* (4.8% to 5.7%). Hep, Int, and Sto of VcHA shared Rhodobacteraceae (16.4%, 8%, and 15.6%, respectively) and Thalassobius sp. (6.2%, 3%, and 5.7%), although *V. shilonii* was the most frequent in Int (25.4%) (Supplementary Table S3).

**Figure 4:**
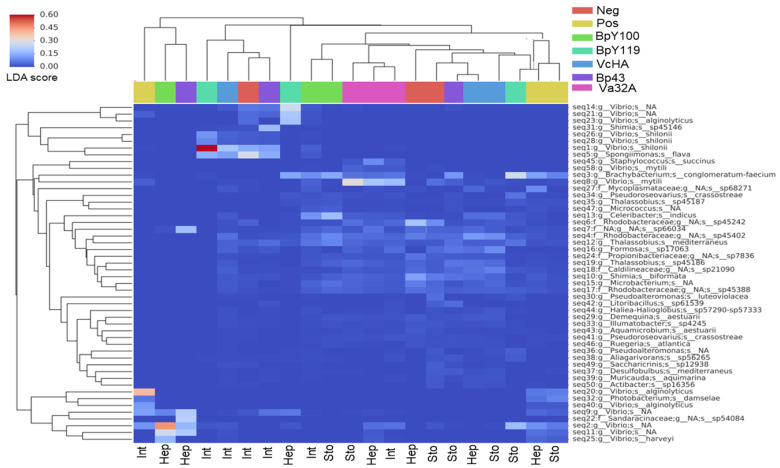
The ASV heatmap shows the abundance of unique amplicon sequence variants (ASV) of each bioassay with probiotics and controls. The top fifty most abundant genera are shown with an LDA score of 0.2 to 0.8.

The ASV heatmap showed the abundance of unique amplicon sequences of each bioassay, with the top fifty most abundant genera. The heatmap shows *Vibrio* (different sequences of *Vibrio* sp., *V. shilonii*, *V. mytili*, and *V. harveyi*), *Shimia*, and *Spongiimononas*, with the maximum LDA score (0.2% to 0.8%) (Figure 4). Due to the presence of *Vibrio* as the most abundant ASV, Spearman’s rank correlation and Kruskal–Wallis tests between Shannon diversity and *Vibrio* abundance were performed; both tests confirmed that the *Vibrio* spp. abundance was significantly lower (*p* < 0.001) in the EP groups compared to the Pos and NEP groups.

### 3.2. Principal Coordinates Analysis with Bray–Curtis Distance

**Figure 5:**
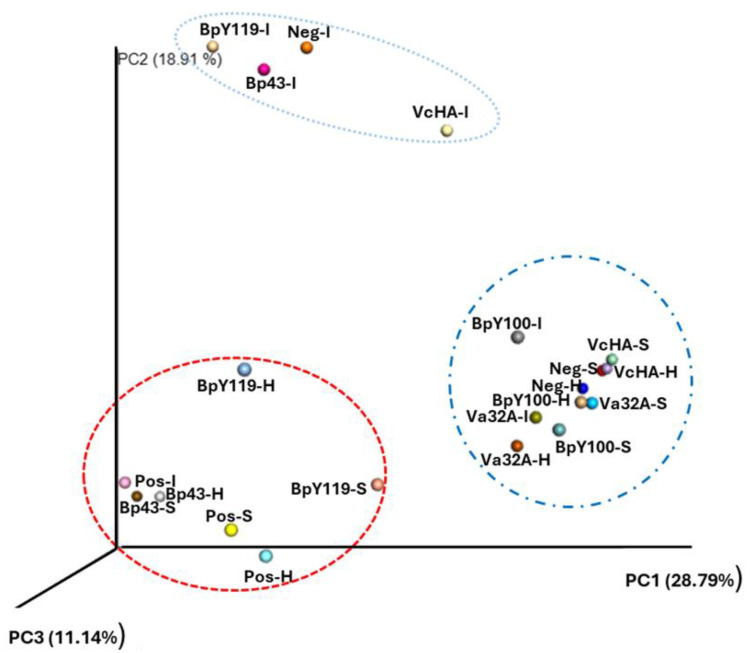
Plot of beta diversities of the microbiome of intestine (I), stomach (S), and hepatopancreas (H) of bioassays with probiotics and positive (Pos) and negative (Neg) controls. Principal coordinates (PC1–PC3) depicting differences and taxonomic composition between the bacterial communities, using unique amplicon sequence variants (ASV). Most samples of effective probiotics (EPs) VcHA, Va32a, BpY100, and Neg control were clustered in the same group due to their taxonomic similarities.

The three-dimensional principle coordinate analysis (PCoA) plot created using the matrix of paired-wise distance between samples calculated by the Bray–Curtis dissimilarity (Beta diversity) was used to compare the community structure among treatments with the unique amplicon sequence variants (ASVs) and revealed that the 21 samples of Int, Sto, and Hep of shrimp infected with Vp_AHPND_/probiotic-fed and controls (Neg and Pos) were grouped in three different clusters, indicating the degree of microbiome similarity. The microbiome of Pos (Hep, Int, and Sto) clustered together with BpY119 (Hep and Sto), and Bp43 (Hep and Sto) (PCo2 = 11.14%). A second cluster grouped a community structure of Int-Neg, with intestinal samples of BpY119, Bp43, and VcHA (PCo2 showed a variation of 18.91%). The third cluster grouped Neg (Sto and Hep) controls with Va32A (Int, Sto, and Hep), BpY100 (Int and Sto), and VcHA (Sto and Hep). PCo1 explained 28.79% of the variation (Figure 5). Moreover, analyses of the relative frequency indicated that the core microbiome of VcHA, Va32A, and BpY100 was mainly represented by *Rhodobacteraceae*, Caldilineaceae, *Celeribacter indicus*, *Illumatobacter*, *Microbacterium*, *Ruegeria atlantica*, *Saccharibacteria* sp., *Shimia biformata*, and *Thalassobius mediterraneus* (Supplementary Table S3).

### 3.3. Biomarkers

The taxa with distributions among different groups were significantly (*p* < 0.05) different and an effect size (LDA score) higher than two was identified with the LEfSe analysis. The positive control showed 32 ASVs, mainly *Photobacterium*, *Photobacterium damselae*, *Fusibacter* sp31471, Colwelliaceae, and Thalassotalea. The negative controls (with 37 biomarkers) showed Rhodobacteraceae, *Pseudoalteromonas luteoviolacea*, *Ruegeria atlantica*, *Tenacibaculum*, and *Illumatobacter*. In the probiotic BpY119 (with eight biomarkers) were Actinobacteria, *Thalassobius*, and *Nitratireductor aquibiodomus*. Probiotic Y100 had eight biomarkers and *Bacillus altitudinis*, *B. pumilus*, *B. safensis*, Sporichthya, and Frankiales possessed the highest LDA scores. The VcHA probiotic had the highest number of biomarkers with 52 and Demequinaceae, Acidimicrobiia, Acidimicrobiales, Planctomycetacia, and Verrucomicrobiaceae had the highest LDA scores. Furthermore, probiotic Va32 A had 13 biomarkers and Bacillales, *Staphylococcus*, Bacilli, *Staphylococcus succinus*, and *Staphylococcus epidermidis hominis* had the highest LDA scores. The complete list of biomarkers with higher LDA scores is shown in Supplementary Figure S1.

## 4. Discussion

A myriad of probiotic applications in the shrimp farming industry has been reported, including the regulation of pond water quality, stimulation of shrimp health status, growth improvement, and prevention and control of diseases [27,28]. In addition, previous studies have compared the gastrointestinal bacterial communities between healthy and diseased (AHPND) and wild or aquacultured *P. vannamei* and *P. monodon* shrimp [10,12,13]. However, research on the profile of the best microbiota diversity in VP_AHPND_-challenged shrimp during probiotic treatments is scarce [20,22].

Our results over the microbiome community showed that the alpha diversity was higher in Sto of almost all trials, which was similar to microbiota reported for the probiotics *Lactobacillus*, *Rhodopseudomonas*, and *EM*. This indicates that microbial diversity may play a crucial role in protecting against bacterial infections; also, the dominant phyla were similar to our study with Proteobacteria and Bacteroidetes [20].

The alpha diversity among the Pos and Neg controls did not show a significant difference according to Tukey’s HSD test. This result is different from that obtained in shrimp fed with ILI probiotics and challenged with Vp_AHPND_ strain BA94C2, where a loss of diversity was associated with AHPND. Such differences may be related to the analysis of only the stomach and hepatopancreas by Restrepo [21], since our study also included the intestine, where the diversity markedly decreased (85 ASVs vs. 29 ASVs and Shannon 4.31 vs. 3.004). Nevertheless, it was compensated by the communities of Hep and Sto-Pos, which increased their abundance with several resident members, such as *Photobacterium damsela*, *V. alginolyticus*, and *Vibrio* sp. (Vibrionacea), although they may cause secondary infections because they are opportunistic pathogens in shrimp [11]. In addition, communities of AHPND-affected shrimp with increased bacterial communities, including *Aeromonas taiwanensis*, *Simiduia agarivorans*, and *Photobacterium angustum*, have been observed [12]. In the present study, Vp_AHPND_ did not decrease the bacterial diversity in Sto of Pos (Shannon index in Pos = 5.68; ASVs = 144; in Neg = 5.62 with 125 ASVs), which was similar to a previous study [10] of the gut bacterial community in AHPND-infected *P. vannamei* whose results agreed with our observations, since the microbiome declined. Furthermore, we showed that the bacterial composition of Pos and Neg controls was significantly different, and the compositional shifts in the gut microbiota of Vp_AHPND_-infected shrimp were enriched with genera *Photobacterium* and *Vibrio*. In contrast, several reports have shown that the intestine of AHPND-infected shrimps had significantly higher bacterial diversity than that of healthy shrimps, whereas in the hepatopancreas, we observed a lower diversity [12,29].

Challenges with probiotics-fed shrimp (BpY100, BPY119, VcHA, Bp43, and Va32A) and those infected with Vp_AHPND_ showed significant microbial diversities. In particular, the Shannon analysis demonstrated a higher alpha diversity in shrimp treated with VcHA probiotics (Figure 2c), which was confirmed by other metrics such as Chao1, PD, and the number of ASVs (Figure 2a,b,d). Different changes in the microbial community structure by probiotic feeding were observed in VpAHPND-challenged shrimp fed with ILI (*Vibrio diabolicus*) and *Bacillus* P64, where the most abundant phyla were Proteobacteria, Tenericutes, Firmicutis, and Cyanobacteria [21], whereas in our study we showed a low abundance of Tenericutes, Firmicutis, and Cyanobacteria (0.1% to 5.1%). In addition, an important reduction in Bacteroidetes (Hep, Int, and Sto) occurred in almost all probiotic trials, except VcHa, and Saccharibacteria in samples treated with BpY119, whereas Actinobacteria showed an increase in abundance in Int and Sto.

Microbiome composition of our negative controls (eight taxa) showed significant differences from Vp_AHPND_-uninfected shrimps cultured in the laboratory [17], wild-caught and domesticated *P. monodon* [30], and farmed *P. vannamei* [31], which indicate than classes, orders, and the family composition of microbial communities are influenced by many particular differences in shrimp farming [8,17,32] and feeding [9,33].

In descending cumulative abundance, Vibrionales (Gammaproteobacteria) > Rhodobacterales (Alphaproteobacteria) > Micrococcales (Actinobacteria) > Flavobacteriales (Bacteroidetes) were reported in our results as the most abundant orders in general, which are different from those reported with ILI or *Bacillus* probiotics, which increased the abundance of Pseudoalteromonadacea (Alteromonadales), Acetobacteracea (Rhodospirillales), and Mollicutes Enterobacteriacea (Enterobacterales) [21]. The latter one was not observed in our results. The presence of Vibrionales is highlighted in Pos and Rhodobacterales in Neg-BpY100 and the abundance of Vibrionales was increased in Hep and Int of BpY119 and Hep of Bp43, which agrees with reports for Hep and Sto with Illi and *Bacillus* probiotics. However, the microbiome of Int was not described by Restrepo [21].

In the present study, Bp43 (Hep) and BpY119 (Hep and Sto) affected bacterial communities. Beta diversity analysis of microbiota clustered them together with the Pos control (PCo3 = 11.14%), where the microbiome diminished by AHPND infection led to dysbiosis (dysbacteriosis), a reduction of beneficial or commensal microbes (11). NEP caused higher mortality of 70% and 100% in previous challenges and did not show any beneficial effect against VP_AHPND_ [23]. This result indicates that not all *B. pumilus* strains should be considered beneficial. Some strains cause diseases in humans and plants; for instance, ginger–rhizome–rot pathogen [34], foodborne illness [35], and cutaneous infection in humans associated with toxicity of *B. pumilus* strains [36], since those strains possess genes encoding virulence factors associated with pathogen–host interactions and the secretion of toxic products [37] because there are also NEP strains of the same species, although the importance of other beneficial *B. pumilus* strains in agriculture and industry is well known [38].

In contrast, the principal coordinate analysis (PCo1 = 28.79) of the microbiota of Va32A (Int, Hep, and Sto), BpY100 (Int, and Sto), and VcHA (Hep, and Sto) showed a clear grouping together with Neg (Sto and Hep) in our study. They had a better protective role against Vp_AHPND_, with the lowest cumulative mortality of 4.76%, 12.54%, and 16%, respectively, in previous challenges and the histopathological and qPCR results were negative [23]. In addition, the effectiveness of EP was statistically confirmed with a low abundance of *Vibrio* spp. (*p* < 0.001), compared with Pos and NEP, supporting the protective role of probiotics in suppressing such pathogens. It has been reported that probiotic treatments with *Lactobacillus* and *Rhodopseudomonas* causing lower survival and showed a higher relative abundance (>60%) of Vibrionaceae in the shrimp gut compared with treatments with higher survival. This also demonstrated that treatments showing higher survival possessed higher Shannon index values (4.69 ± 0.133) compared with those with lower survival [20], since protective strains of *B. pumilus* produce anti-*Vibrio* substances, structurally identical to amicoumacin, involved in the formation of cell cavities and the disruption of cell membranes [39]. We highlighted the core microbiome of EP VcHA, where *Ruegeria* and *Shimia* have been described as beneficial bacteria that participate in phosphate and protein metabolism [40]. Specific taxa from Rhodobacteraceae and Microbacteriaceae may efficiently resist the colonization of *Vibrio* in shrimp guts by improving the gut bacterial community stability or secreting antibacterial substances [20], and *Microbacterium* sp. also serves as an edible compliment or synthesizes nutrients that promote host growth, such as vitamins [41]. The highest abundance of Rhodobacteraceae bacteria in these probiotics may indicate that they display probiotic potential due to their potential to synthesize vitamin B12, which is required for shrimp growth, and produce substances to inhibit pathogens (tropodithietic acid; TDA) [42]. Notably, this family is a regular inhabitant of the stomach [16], as in the core microbiome of shrimp fed with probiotics VcHA, Va32A, and BpY100.

The principal coordinate analysis showed that these samples clustered separately according to treatments, indicating that community structure composition and diversity among groups differed based on the Bray–Curtis distance [18]. The linear discriminant analysis (LDA) using LEfSe showed the taxa more abundant in biomarkers with the highest LDA score in the probiotic Va32A were Firmicutes and *Staphylococcacea* (*S. succinus*, *S. epidermidis*). In VcHA, they were Demequinacea, Acidomicrobia, and Planctomyces; and in BpY100, they were Bacillacea and *B. altitudinis-pumilus*. However, no previous reports on similar studies with LEfSe analyses were found, which indicates that this is the first study in this field. Our results only agreed with the biomarkers Rugeria and Vibrionales in healthy and AHPND-infected shrimp [11,21].

Our study possesses several limitations. We did not include an adequate sample size in the experimental design, thus low sample sizes are unlikely to capture the individual variation often associated with microbiome surveys that may affect an accurate description of the distribution of data and statistical testing [43]. Due to the limitations of 16S rRNA gene sequencing and despite being a fast and easily accessible testing method for biomarker discovery, together with the proper limitations of the reference databases, 0% to 5.5% of some species were not detected in our samples (cited as None: other in Supplementary Tables S1–S3) of the microbiome, which is also a limitation for a correct and complete identification of all biomarkers via LEfSe [44]. Furthermore, our study lacks an untreated, unchallenged probiotic-fed control, which may clarify whether microbiome shifts result from disease status, probiotic supplementation, or their interaction among native gut microbiota and probiotic bacteria [45].

## 5. Conclusions

This study is the first to evaluate the Int, Sto, and Hep bacterial communities in *P. vannamei* infected with Vp_AHPND_ and fed probiotics. Proteobacteria (Gammaproteobacteria), Actinobacteria, Bacteroidetes, and Saccharibacteria were the most abundant phyla among the Hep, Int, and Sto. In addition, alpha diversity was higher in almost all trials of EPs (VcHA, Va32A, and BpY100), which may indicate that microbial diversity plays a crucial role in protecting against bacterial infections, since high diversity was statistically associated with low mortality. Although diversity markedly decreased in the Int, it was increased in the Hep and Sto-Pos. Alphaproteobacteria was the most common in the Hep and Sto of the Neg control, but this was displaced by Gammaproteobacteria in Vp_AHPND_-infected shrimp (Pos). Each probiotic caused changes in the abundance of both classes, including Bacteriodetes and Actinobacteria. At the order level, Vibrionales (Gamaproteobacteria), Rhodobacterales (Alfaproteobacteria), and Micrococcales (Actinomycetes) were the most prominent in almost all trials. Beta-diversity analysis clustered microbiome similarity among challenges with shrimp treated with the best probiotics VcHA, Va32A, and BPY100. These results are consistent with previous bioassays from which samples were obtained for metagenomic analyses. The ASV heatmap and analyses of relative frequency at the genera and species level identified the core microbiome of EP, composed of *Rhodobacteraceae*, Caldilineaceae, *Celeribacter indicus*, *Illumatobacter*, *Microbacterium*, *Ruegeria atlantica*, *Saccharibacteria* sp., *Shimia biformata*, and *Thalassobius mediterraneus*. The highest abundance of Rhodobacteraceae and *Microbacterium* in these probiotics may indicate that they display probiotic potential because of the synthesis of complementary nutrients and vitamin B12, required for shrimp growth, and essential compounds to inhibit pathogens, such as TDA. The protective effect of probiotics must not only be attributed to microbiome shifts, but also the host-driven microbial selection and their interactions with native gut bacteria, which strengthen the development of the host immune and digestive system to protect shrimp from harmful microorganisms, should be considered. The use of feed supplemented with probiotics can effectively promote the growth and survival of beneficial bacteria in the gut for the development and maintenance of a healthy gut microbiome, but there is still much to learn about its complex interactions, which will allow us to gain new insights and generate more data as the field grows. Our results present a sustainable and ecosystem-friendly alternative in place of antibiotics, based on beneficial microorganisms to prevent and control AHPND, and probably other bacterial diseases, in shrimp farming.

## Figures and Tables

**Table 1:** Raw sequences and number of sequences obtained from the five different probiotics per trial and controls.

Trial	Organ *	Raw seq (R1 + R2)	Trimmed seq (R1 + R2)	Chimera Free seq	N° of ASVs Per Organ	Total of ASVs Per Trial	SRA Accession Number
Neg	Hep	58,274	57,950	24,484	83		SAMN37458684
	Int	66,018	65,680	28,116	85	293	SAMN37458685
	Sto	52,442	52,104	22,359	125		SAMN37458686
Pos	Hep	45,648	45,310	18,993	108	281	SAMN37458689
	Int	45,944	45,702	15,638	29		SAMN37458687
	Sto	56,104	55,710	22,437	144		SAMN37458688
BpY100	Hep	49,354	49,028	19,549	16		SAMN37458690
	Int	47,772	47,468	20,018	117	223	SAMN37458691
	Sto	66,502	66,060	26,552	90		SAMN37458692
BpY119	Hep	62,882	62,490	27,985	60		SAMN37458693
	Int	59,734	59,390	26,789	37	213	SAMN37458694
	Sto	63,434	62,982	27,791	116		SAMN37458695
VcHA	Hep	55,020	54,558	23,941	166		SAMN37458696
	Int	70,452	70,018	30,760	136	449	SAMN37458697
	Sto	66,192	65,638	28,416	147		SAMN37458698
Bp43	Hep	73,700	73,050	31,367	7		SAMN37458699
	Int	43,668	43,474	18,092	79	196	SAMN37458700
	Sto	35,834	35,626	15,612	110		SAMN37458701
Va32A	Hep	40,460	40,244	17,647	87		SAMN37458702
	Sto	36,666	36,484	15,773	78	275	SAMN37458703
	Int	38,996	38,788	16,680	110		SAMN37458704
	Total	1,135,096	1,127,754	478,999	1930	1930	

Abbreviations: Neg = Negative control, Pos = Positive control, Seq = Sequences, * Hep = Hepatopancreas, Int = Intestine, Sto = Stomach, ASVs = Amplicon sequence variants.

**Table 2:** Values of relative abundance (percent) of the four most abundant phyla for shrimp treated with probiotics, negative control and shrimp inoculated with Vp-AHPND (positive control).

Trial	Organ	Phylum Level			
		Actinobacteria	Bacteroidetes	Proteobacteria	Saccharibacteria
Neg	Hep	23.0%	0.1%	60.4%	5.5%
	Int	2.9%	33.2%	56.8%	0.4%
	Sto	11.6%	17.0%	63.4%	4.0%
Pos	Hep	17.7%	2.0%	61.8%	1.6%
	Int	0.3%	0.0%	99.6%	0.0%
	Sto	10.6%	3.0%	72.5%	1.0%
BpY00	Hep	0.0%	0.1%	98.3%	1.1%
	Int	22.2%	6.3%	57.3%	3.4%
	Sto	31.8%	0.2%	58.8%	5.3%
Bp119	Hep	14.2%	0.1%	83.7%	0.8%
	Int	0.7%	15.1%	83.3%	0.1%
	Sto	30.7%	1.9%	63.4%	1.1%
VcHA	Hep	12.8%	13.7%	48.7%	6.8%
	Int	7.1%	20.6%	62.6%	1.9%
	Sto	14.6%	16.3%	53.3%	3.6%
Bp43	Hep	0.0%	0.0%	82.0%	18.0%
	Int	1.7%	18.7%	68.5%	0.4%
	Sto	23.0%	9.4%	58.0%	3.4%
Va32A	Hep	13.8%	3.2%	59.5%	6.6%
	Int	13.7%	11.3%	64.4%	1.7%
	Sto	16.0%	3.8%	72.1%	2.0%

Abbreviations: Neg = Negative control, Pos = Positive control.

## Data Availability

Raw data were stored in the sequence read archive (SRA), NCBI GenBank, in the Bioproject PRJNA1018963 under the accession numbers SAMN37458684, SAMN37458685, SAMN37458686, SAMN37458689, SAMN37458687, SAMN37458688, SAMN37458690, SAMN37458691, SAMN37458692, SAMN37458693, SAMN37458694, SAMN37458695, SAMN37458696, SAMN37458697, SAMN37458698, SAMN37458699, SAMN37458700, SAMN37458701, SAMN37458702, SAMN37458703, and SAMN37458704.

## Supplementary Materials

The following supporting information can be downloaded at: https://www.mdpi.com/article/10.3390/pathogens14040320/s1, Table S1. Relative frequency (%) of bacterial composition at phylum level observed among five trials of shrimp fed with probiotics and infected with Vp-AHPND. Table S2. Relative frequency (%) of the main bacterial class of shrimp fed with probiotics and challenged with VP_AHPND_. Table S3. Relative frequency (%) of microbiome composition at genera/species level of five probiotic treatments and controls. Figure S1. The complete list of biomarkers with a higher LDA score.
