# Supplementary figures and images for: Characterization of Microbiome Diversity in the Digestive Tract of Penaeus vannamei Fed with Probiotics and Challenged with Vibrio parahaemolyticus Acute Hepatopancreatic Necrosis Disease
Source: Pathogens. 2025 Mar 27;14(4):320. doi: 10.3390/pathogens14040320 (PMC12030750; doi:10.3390/pathogens14040320)

Biomarkers Ordered by Effect Size (LDA Score)

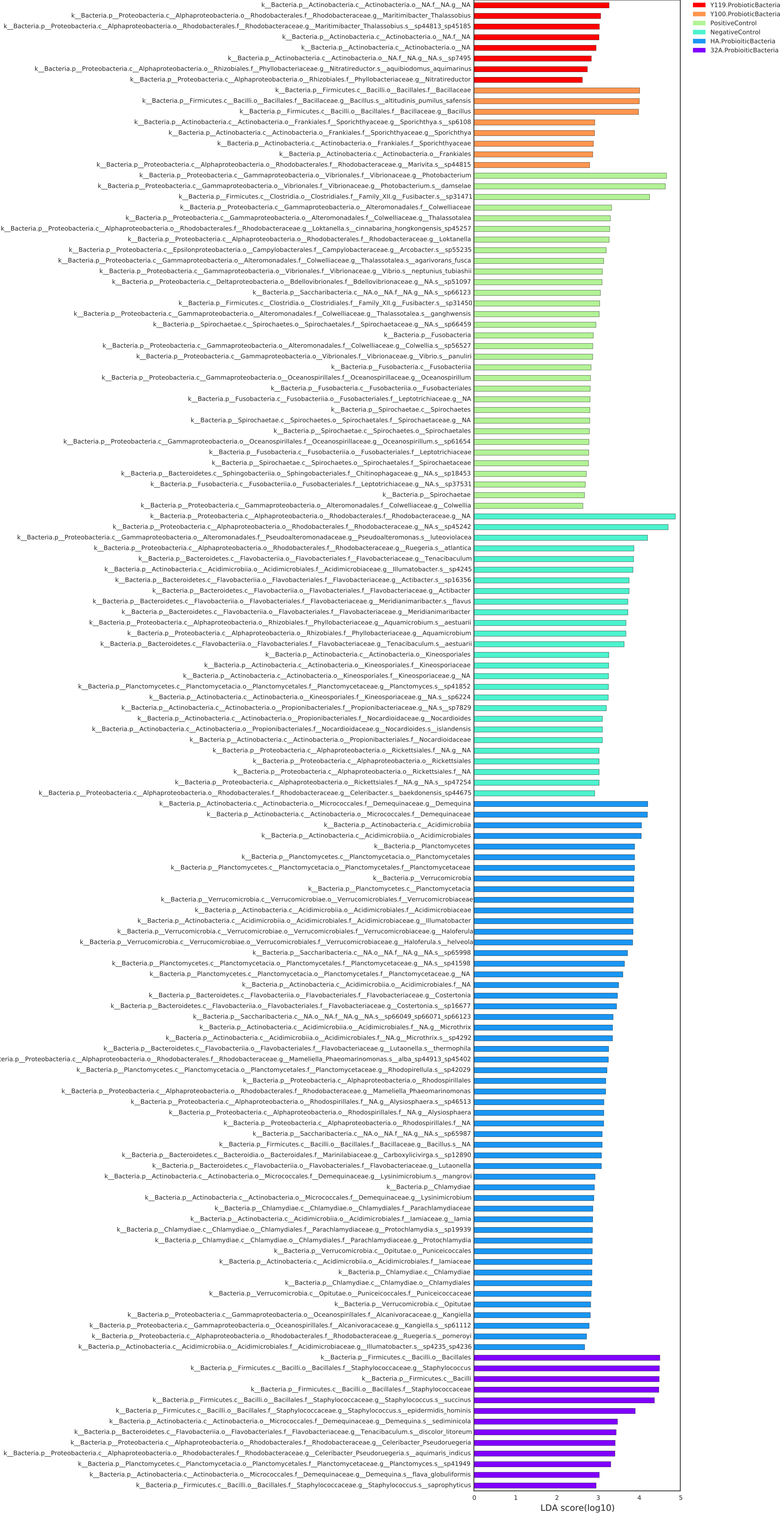

Supplement: Supplementary file 1 [file pathogens-14-00320-s001.zip › pathogens-3503033-Figure S1.pdf]
